# Ferritin Cage–Alginate Oligosaccharide-Stabilized Emulsion for Co-Carriage and Protection of Zinc Ion and Hydrophobic Molecule

**DOI:** 10.3390/foods15101666

**Published:** 2026-05-11

**Authors:** Jincan Wu, Yumeng Zhang, Ruge Cao, Shihao Sun, Rui Yang

**Affiliations:** 1 Application Deparment, Beijing Life Science Academy, Beijing 102209, China; 2 State Key Laboratory of Food Nutrition and Safety, College of Food Science and Engineering, Tianjin University of Science & Technology, Tianjin 300457, China; 3 Public Health College, Xuzhou Medical University, Xuzhou 221004, China

**Keywords:** ferritin, zinc ions, alginate oligosaccharide, emulsion, astaxanthin

## Abstract

Ferritin, a natural cage-like protein, can be applied as a nanomaterial to encapsulate and deliver bioactive ingredients, while challenges remain when using ferritin to deliver multiple bioactive ingredients. In this study, a ferritin–zinc ion–alginate oligosaccharide (AOS) core–shell complex (FZA) and hydrophobic astaxanthin (AST) were applied as the water and oil phase to prepare oil-in-water emulsions simultaneously containing mineral element and hydrophobic AST. The ferritin works as a multicompartment carrier to encapsulate the Zn^2+^ ions and bind with the AOS. This emulsion exhibited smaller particle size and higher apparent viscosity, elastic modulus, and anti-delamination stability. After heat treatment, natural light irradiation, and ultraviolet irradiation, the retention rates of AST in FZA-stabilized emulsion were increased by 23.09%, 18.25%, and 19.24%, respectively, compared with AST dissolved in oil. The release rate of AST in FZA-stabilized emulsion was increased by 26.97% compared with that dissolved in oil in vitro digestion simulation, and release rate of Zn^2+^ ions in FZA-stabilized emulsion improved by 20.38% relative to the control. This study provides experimental evidence for the emulsion stabilized by the AOS and ferritin multi-interface, which achieves dual co-delivery and protection of mineral and hydrophobic molecules.

## 1. Introduction

Ferritin, a natural cage-like protein, can be applied as a nanomaterial to encapsulate and deliver bioactive ingredients; the use of ferritin’s special cage-like structure can improve the stability of bioactive molecules [1], and it has been shown that encapsulating bioactive ingredients such as epigallocatechin gallate and rutin into the interior of ferritin not only improved the stability of the bioactive molecules, but also allowed for the controlled release of the molecules in a more sustained manner [2,3]. In addition to this, by combining ferritin with mineral elements, the disadvantages of low solubility of mineral elements and their susceptibility to the environment of the gastrointestinal tract can be overcome [4,5]. However, existing research has predominantly centered on encapsulating individual bioactive compounds, and the utilization of ferritin as a delivery vehicle for multiple bioactive agents simultaneously remains challenging.

Early studies have shown that natural ferritin could use as an emulsifier, suggesting that ferritin has emulsifying capabilities [6,7]. However, emulsions stabilized by proteins often exhibit limited stability due to multiple factors. Electrostatic repulsion between oil droplets, governed by the net charge of interfacial proteins, weakens when the pH approaches the protein’s isoelectric point, thereby promoting droplet flocculation or coalescence [8]. Moreover, the interfacial protein layer’s thickness and structural integrity can be altered by pH, ionic strength, and temperature. These factors collectively contribute to the instability of protein-stabilized emulsions [9,10]. Alginate oligosaccharide (AOS) is an anionic oligosaccharide derived from sodium alginate [11]. It has been shown that anionic polysaccharides can form a negatively charged interfacial film around emulsion droplets, and reduce droplet aggregation, leading to greater emulsion stability [12]. However, the application of the multilayer emulsion system stabilized by ferritin–anionic oligosaccharides in the co-delivery of hydrophobic substances and mineral elements has not yet been clarified.

Zinc ions, as a necessary mineral element for the human body, play extremely important roles in human growth and immunity functions [13]. Astaxanthin (AST) is a potent fat-soluble antioxidant with various nutritional benefits and can act as a colorant in food [14]. However, AST is easily decomposed under heat and light conditions, which limits its application. In addition, when AST and Zn^2+^ ions are present together, their antioxidant activity can be enhanced [15]. In our previous study, the structural characteristics of ferritin were used to encapsulate AST and Zn^2+^ ions together in the cavity of ferritin, and AOS was used to form a nanoparticle complex, which was able to effectively protect AST and Zn^2+^ ions, and significantly enhanced the antioxidant activity of AST [16]. The aim of this study is to build a ferritin-based multilayer emulsion delivery system, in order to achieve efficient co-encapsulation and protection of AST and Zn^2+^ ions. Distinct from the previous study, a ferritin–zinc ion–alginate oligosaccharide complex (FZA)-based emulsion system was successfully constructed in this work, achieving the co-encapsulation and delivery of a hydrophobic bioactive compound together with a mineral element for the first time. The FZA acted as a particulate emulsifier to stabilize the oil–water interface, forming an emulsion with a dense network structure. The regulatory mechanisms of FZA in the emulsion formation process (including droplet size, interfacial charge, rheological behavior, and storage stability) were systematically elucidated. Moreover, the protective effects of this multilayered emulsion system on both AST and Zn^2+^ ions were demonstrated. In addition, the contribution of Zn^2+^ ions to tuning the interfacial and rheological characteristics was explored, which provided a reference for application of ferritin-stabilized emulsion in co-delivery of a variety of bioactive compounds.

## 2. Materials and Methods

### 2.1. Materials

Ferritin expression strains were retained in the laboratory. Alginate oligosaccharide (an average molecular weight of 2100 Da) was purchased from Qingdao BZ Oligo Biotech Co., Ltd. (Qingdao, China). Pepsin and trypsin were purchased from Dalian Meilunbio Biotechnology Co., Ltd. (Dalian, China). Astaxanthin was purchased from Shanghai Xushuo Biotechnology Co., Ltd. (Shanghai, China). All chemicals used in this study were analytical-grade chemicals.

### 2.2. Preparation of Ferritin and Ferritin–Zinc Ion–Alginate Oligosaccharide Complex

Ferritin was extracted according to a previously reported method [17]. To prepare the ferritin–zinc ion complex (FZ), ferritin solution was mixed with ZnSO_4_ solution to make mass ratios of 1:50, 1:70, 1:90, 1:110, and 1:130 (ferritin/ZnSO_4_). The mixture was stirred for 30 min, after which free Zn^2+^ ions were removed by dialysis in Tris-HCl buffer using a 10 kDa cutoff dialysis bag. The ferritin–AOS complex (FA) was prepared by adding AOS solution to ferritin to make a mass ratio of 1:80 (ferritin/AOS), followed by stirring for 30 min. The ternary complex (FZA) was then obtained by adding AOS to the preformed FZ complex at the same ferritin-to-AOS mass ratio of 1:80. In short, the 100 μL sample to be tested was added to 675 μL GdnHCl solution (8 M) for 30 min to dissociate ferritin and release Zn^2+^ ions. Subsequently, 225 μL of 0.8 mM [4-(2-pyridinylazo)resorcinol] (PAR) was introduced [18]. The Zn^2+^ ion concentration was determined by measuring absorbance at 500 nm and interpolating from a standard curve (y = 0.0026x + 0.141, R^2^ = 0.9946).

### 2.3. Characterization of Ferritin–Zinc Ion–Alginate Oligosaccharide Complex

#### 2.3.1. Ultraviolet–Visible Absorption and Fluorescence Spectrum Analysis

UV–vis absorption spectra were recorded on an Agilent 8453 spectrophotometer over a wavelength range of 200–400 nm. Fluorescence emission spectra were measured with a fluorescence spectrophotometer (Agilent, Santa Clara, CA, USA) at an excitation wavelength of 290 nm, with emission scanned from 300 to 500 nm. Both excitation and emission slit widths were set to 5 nm.

#### 2.3.2. Zeta-Potential and Interaction Force Analysis

Zeta-potential was measured using a Zetasizer Advance (Malvern, London, UK). Each sample was equilibrated within the instrument for 60 s prior to analysis, and at least three consecutive readings were recorded. According to the previous method, the relative strength of the molecular interaction between ferritin, Zn^2+^ ions, and AOS was determined [19,20,21,22,23]. In short, 0.1 M NaCl was added to FZ, FA, and FZA solutions to shield electrostatic interaction. Absorbance of the samples at a wavelength of 600 nm and relative strength of electrostatic interaction were measured by difference in absorbance before and after NaCl was added. Similarly, 0.1 M urea and 0.1 M SDS were used to evaluate the relative strength of hydrogen bond and hydrophobic interaction.

### 2.4. Preparation of Emulsion

Emulsion was obtained by adding 20% soybean oil to the FZ, FA, and FZA solutions prepared in Section 2.2 and homogenizing at 12,000 rpm for 2 min. AST-containing soybean oil was prepared by adding AST (3 mg) to soybean oil (10 mL) and stirring continuously at 45 °C for 10 min to obtain AST–soybean oil (AST-Oil). The FZ-stabilized emulsion (without AST) was labeled as FZ-E; the FA-stabilized emulsion (without AST) was labeled FA-E; the FZA-stabilized emulsion (without AST) was labeled FZA-E; the FZ-stabilized emulsion (including AST) was labeled FZ-AE; the FA-stabilized emulsion (including AST) was labeled FA-AE; and the FZA-stabilized emulsion (including AST) was labeled as FZA-AE.

### 2.5. Emulsifying Properties

To investigate the influence of molar ratio of ferritin to AOS on emulsifying properties, AOS was added to ferritin at mass ratios of 1:0, 1:20, 1:40, 1:60, 1:80, and 1:100. For this, 20% (*v*/*v*) soybean oil was added, and the preparation method of the emulsion referred to in Section 2.4 was used. In addition, the ESI and EAI of the FZ-E, FA-E, FZA-E, FZ-AE, FA-AE, and FZA-AE were also determined.

The emulsification properties were measured using the previous method with a slight modification [24]. In short, the emulsion obtained after 0 and 10 min was diluted 50 times with 0.1% (*w*/*v*) SDS solution. Then, absorbance at 500 nm was measured with the 8453 Agilent spectrophotometer (USA). The emulsifying activity index (EAI) and the emulsifying stability index (ESI) were calculated by Equations (1) and (2):

(1) $$EAI\left(m^{2}/g\right)=\frac{2\times 2.203\times A_{0}\times DF}{C\times (1-\varphi )\times 10,000}$$

(2) $$ESI\left(\%\right)=\frac{A_{10}}{A_{0}}\times 100$$

where A_0_ and A_10_ are absorbance of 0 and 10 min after homogenization, DF is dilution factor (50), φ is fraction of oil (0.2), and C is protein concentration (g/mL).

### 2.6. Zeta-Potential, Particle Size, and Polydispersity Index of Emulsions

The homogenized emulsion was diluted 100 times, and then analyzed with a zetasizer advance (Malvern, UK). The polydispersity index (PDI) was measured with a nanometer particle size analyzer (Dandong Baiter, Dandong, China) at 25 °C. Each sample was equilibrated within the instrument for 60 s prior to analysis, and at least three consecutive readings were recorded.

### 2.7. Optical Microstructure

Optical microstructure of the emulsion was observed using an optical microscope (Leica, DM1000 LED, Wetzlar, Germany) and intelligent high-resolution microscopic fast imaging (Axio imager M2, Zeiss, Oberkochen, Germany). After the emulsion was diluted by Tris-HCl (pH 7.5), the oil and protein were stained by adding 0.1% (*w*/*v*) Nile blue and 0.1% (*w*/*v*) Nile red to samples, respectively. Stained images were captured at excitation wavelengths of 488 and 633 nm.

### 2.8. Stroage Stability of Emulsions

Emulsion storage stability was assessed by creaming index (CI) [25]. Specifically, fresh emulsion was stored at 4 °C. After 7 days of storage, the CI value was recorded and measured by Equation (3).

(3) $$CI\left(\%\right)=\frac{H_{t}}{H_{0}}\times 100$$

where H_t_ and H_0_ are height of serum layer and total emulsion, respectively.

### 2.9. Rheological Properties of Emulsions

Emulsion was loaded onto a dynamic rheometer (HAAKE MARS 60, Thermo Fisher Scientific, Waltham, MA, USA) and the steady-state shear experiment and dynamic sweep experiment were performed on the emulsion respectively; the experimental method was determined according to the previous method [26]. Storage modulus (G′) and loss modulus (G″) were measured with frequency rate at 1% strain. Fresh emulsion (200 μL) was loaded onto the parallel plates, and apparent viscosity (η) was measured as shear rate rose from 0.1 to 10 s^−1^. The emulsions followed the Ostwald–de Waele power-law model [27].

### 2.10. Encapsulation Efficiency and Stability of AST in Emulsion

#### 2.10.1. Encapsulation Efficiency

The emulsion was centrifuged (4000 rpm, 5 min), and the lower clear liquid (water phase) and emulsion layer were separated. The emulsion layer was taken and mixed with an equal volume of organic solvent (n-hexane: anhydrous ethanol = 2:1, *v*/*v*), and then centrifuged at 10,000 rpm for 5 min. Supernatant was collected and its absorbance at 480 nm was measured to measure the content of AST, and the encapsulation efficiency was calculated according to Equation (4):

(4) $$Encapsulation\;efficiency\;\left(\%\right)=\frac{Amount\;of\;AST\;in\;emulsion\;layer}{Total\;AST\;added}\times 100$$

#### 2.10.2. Storage Stability

Retention rate of AST in emulsion was determined with a slightly modified method based on the previous research process [28]. Emulsion was stored at room temperature and 4 °C in dark. Every 24 h, samples were taken and mixed with an equal volume of organic solvent (n-hexane: anhydrous ethanol = 2:1, *v*/*v*) to quantify residual AST using Equation (5):

(5) $$Retention\;rate\;of\;AST\;\left(\%\right)=\frac{A_{t}}{A_{0}}\times 100$$

where A_t_ and A_0_ represent the absorbance of the AST after storage and initially, respectively.

#### 2.10.3. Thermal Stability and Photostability

To assess the thermal stability of AST, freshly prepared samples were heated at 95 °C for 30 min, and retention rate of AST was measured. Additionally, the light stability of AST was evaluated by exposing the sample to a 254 nm ultraviolet lamp and natural light at room temperature for 6 h. Processed samples were collected every 2 h and the retention rate of AST was measured.

### 2.11. Simulation of Gastrointestinal Digestion

The in vitro digestion model was established following previously described methods [28,29,30,31,32]. Simulated gastric fluid (SGF) was formulated with 3.2 mg/mL pepsin, 0.7% HCl (*v*/*v*), and 2 mg/mL NaCl, pH 2.0. Emulsion (5 mL) was mixed with SGF and incubated under 37 °C (100 rpm) for 2 h. Digestion was halted by adjusting pH to 7.0. Thereafter, simulated intestinal fluid (SIF) was added to gastric digesta. SIF was made by dissolving 0.68 g K_2_HPO_4_ in 50 mL water, adjusting pH to 6.8 with 1 M NaOH, and supplementing with 3 mg/mL trypsin. The mixture was then shaken for 2 h at identical temperature and speed. After digestion, samples were immediately placed in an ice bath to stop trypsin activity.

After centrifuging the digested mixture at 10,000 rpm for 15 min at 4 °C, the resulting supernatant was taken, mixed with an equal volume of organic solvent (n-hexane: anhydrous ethanol = 2:1, *v*/*v*) and centrifuged at 10,000 rpm for 5 min; n-hexane-phase-dissolved AST and the ethanol-phase-dissolved Zn^2+^ ions were collected. Absorbance at 480 nm of n-hexane phase was used to measure the content of AST. As for the Zn^2+^ ion content, the 100 μL sample to be tested was added to 900 μL 0.2 mM PAR solution [33]. Absorbance at 500 nm was measured to obtain the content of Zn^2+^ ions. Release rate of AST and Zn^2+^ ions was calculated by Equations (6) and (7).

(6) $$Release\;rate\;of\;AST\;\left(\%\right)=\frac{C_{1}}{C_{2}}\times 100$$

(7) $$Release\;rate\;of\;{Zn}^{2+}\;ions\;\left(\%\right)=\frac{C_{3}}{C_{4}}\times 100$$

C_1_ and C_3_ represent contents in supernatant, and C_2_ and C_4_ represent contents in initial emulsion, respectively.

### 2.12. Statistical Analysis

Experimental data were analyzed statistically with SPSS 23.0. Differences among groups were assessed by one-way ANOVA with Duncan’s test. Each experiment was repeated three times for validation, and results are expressed as means ± standard deviation (SD). 

## 3. Results and Discussions

### 3.1. Ferritin Polyacrylamide Gel Electrophoresis (SDS-PAGE) Analysis

**Figure 1:**
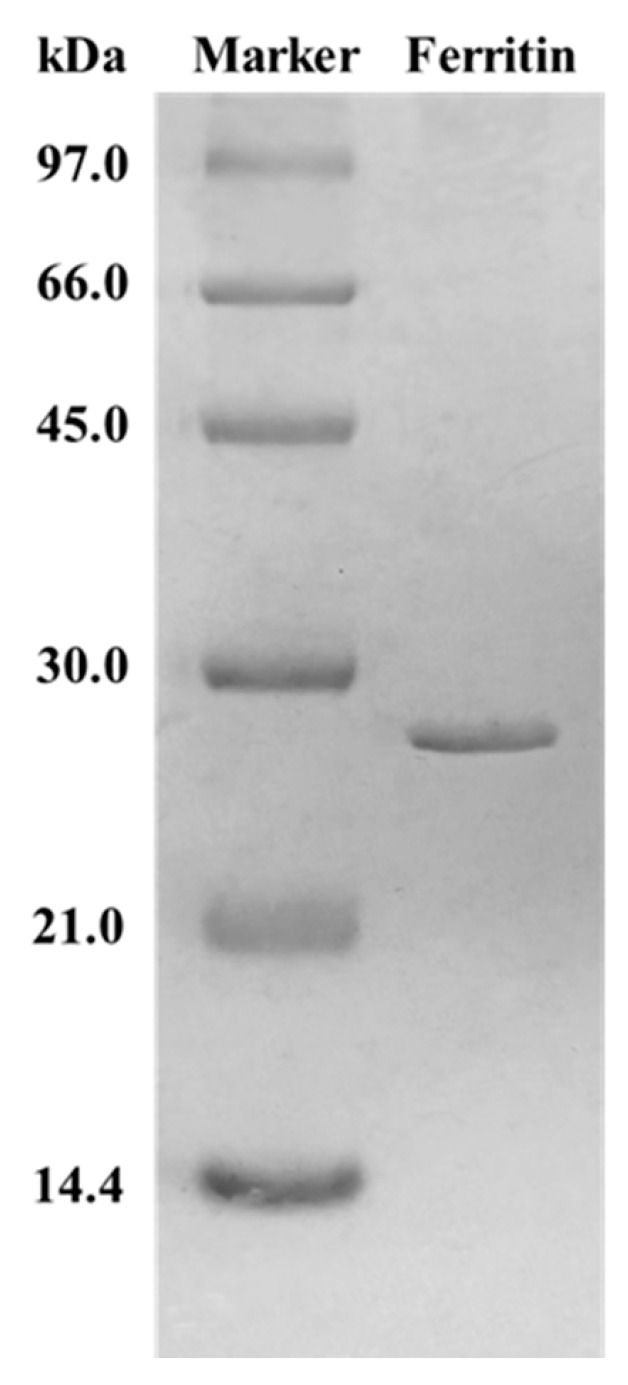
SDS-PAGE analysis of the recombinant H-2-type soy ferritin.

The ferritin used was recombinant H-2-type soy ferritin, which has the same structure as natural H-type soy ferritin. The SDS-PAGE result is shown in Figure 1. The same expression and purification system was used as in our previous study, and the result was consistent with the previous result [16].

### 3.2. Characterization of Ferritin–Zinc Ion–Alginate Oligosaccharide Complex

#### 3.2.1. Ultraviolet–Visible Absorption and Fluorescence Spectrum Analysis

**Figure 2:**
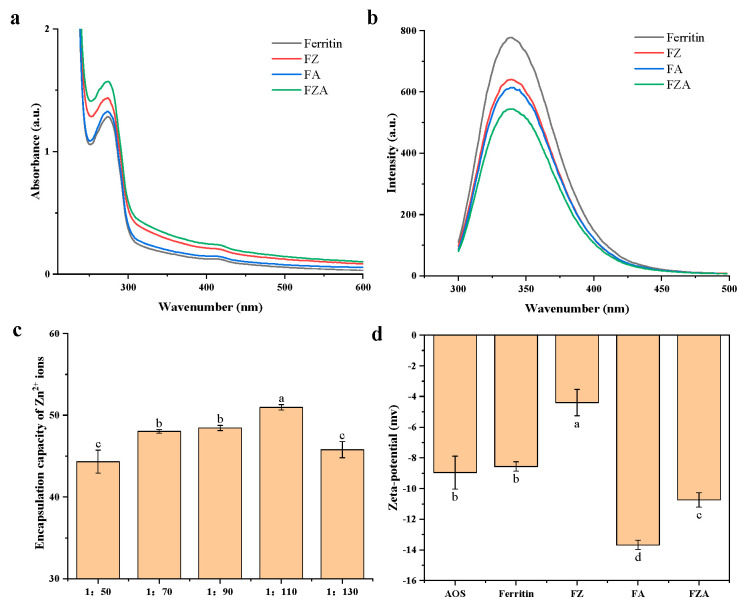
(**a**) Ultraviolet–Visible absorption of ferritin, FZ, FA, and FZA. (**b**) Fluorescence spectrum of ferritin, FZ, FA, and FZA. (**c**) Encapsulation capacity of Zn^2+^ ions. (**d**) Zeta-potential of ferritin, FZ, FA, and FZA. Different letters (a–d) on the column represent statistically significant (*p* < 0.05).

The characteristic absorption peaks of ferritin, FZ, FA, and FZA were at 280 nm (Figure 2a) [4]. The absorbance of FZA at the characteristic absorption peak was enhanced, so we concluded that non-covalent interactions, specifically electrostatic forces and hydrogen bonding, play a major role in the binding of ferritin with Zn^2+^ ions and AOS [34].

The changes in ferritin conformation can be reflected by monitoring changes in the fluorescence intensity of tryptophan at 330 nm (excitation wavelength 290 nm) [35]. As shown in Figure 2b, after binding to Zn^2+^ ions, the fluorescence intensity decreased from 778.4 to 640.0 a.u., indicating that the Zn^2+^ ions entered the cavity of ferritin through electrostatic interactions with changing microenvironment around tryptophan residues, shielding the chromophore on the surface of tryptophan [36]. After being combined with AOS, the fluorescence intensity dropped to 614.0 a.u., indicating that the interaction between AOS and the surface of ferritin could also alter the microenvironment of tryptophan. The attachment of AOS to the surface of ferritin could shield the tryptophan chromophore groups on its surface [16]. In the presence of Zn^2+^ ions and AOS (FZA), the fluorescence intensity decreased to 546.0 a.u., indicating that the Zn^2+^ ions and AOS on the structure of ferritin had a synergistic effect.

#### 3.2.2. Stoichiometry Analysis of the Ferritin and Zn^2+^ Ions

The results showed that when 110 Zn^2+^ ions reacted with ferritin, each ferritin molecule could bind about 50.96 ± 0.33 Zn^2+^ ions (Figure 2c), which was similar to the previous study where each molecule of human H-chain ferritin could bind 48 Zn^2+^ ions [37]. The binding of the Zn^2+^ ions in ferritin provided an ideal space for the delivery of Zn^2+^ ions.

#### 3.2.3. Zeta−Potential and Interaction Force Analysis

At pH 7.5, ferritin and AOS were negatively charged, while the zeta-potential of FZ was higher than ferritin (Figure 2d). This was because of the entry of positively charged metal ions into ferritin, thereby altering the charge distribution on its surface [38]. Compared with ferritin and FZ, the zeta−potentials of FA and FZA decreased from −8.56 mV and −4.39 mV to −13.7 mV and −10.74 mV respectively, which was because the adsorption of AOS to the similarly negatively charged ferritin surface through interactions such as hydrogen bonding reduced the exposure of positively charged groups [39]. The zeta−potential of FZ, FA, and FZA ranged from −4.39 mV to −13.7 mV. Studies have shown that when the zeta-potential of ferritin is close to or lower than −5 mV, it could lead to the aggregation of ferritin [40]. This moderate aggregation trend might help form an interconnected network structure at the oil–water interface, thereby enhancing mechanical strength of interface membrane and providing spatial stability [19]. This phenomenon has been reported in a related study, which found that the electrostatic interaction between myofibrillar protein and chitosan promoted aggregation of protein–polysaccharide complexes, thereby facilitating the construction of a stable emulsion system [41].

Some chemical agents affect the intermolecular interaction and cause changes in the structure of the complex, which can be monitored by absorbance. The relative strengths of interaction forces of FZ, FA, and FZA are shown in Table 1. The electrostatic force played a dominant role in FZ, indicating that ferritin and Zn^2+^ ions were mainly bonded by electrostatic attraction [42]. There were electrostatic interactions, hydrogen bonds, and hydrophobic interactions between ferritin and AOS, which were the key forces for the formation of protein–oligosaccharide complexes [10,12]. In addition, FZA had higher electrostatic force, hydrogen bond, and hydrophobic interaction, which might be due to the interaction between ferritin and Zn^2+^ ions, exposing more positive charges, hydrogen binding sites and hydrophobic groups, thus increasing the interaction force between AOS and FZ.

### 3.3. Emulsifying Properties

**Figure 3:**
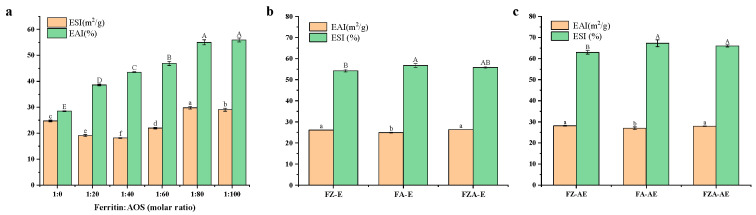
(**a**) The ESI and EAI of emulsions formed by ferritin with different molar ratios of AOS. (**b**) The EAI and ESI of emulsion without AST. (**c**) The EAI and ESI of emulsion with AST. Different letters (a–f/A–E) on the column represent statistically significant (*p* < 0.05).

The ESI and EAI are helpful in determining the reaction ratio of ferritin and AOS to ensure an appropriate amount of AOS addition. As shown in Figure 3a, after the reaction between AOS and ferritin, as the concentration of AOS increased, the EAI of ferritin–AOS complex first decreased and then increased. We speculated that this might be due to the addition of AOS promoting the formation of protein polymers, reducing the water-holding capacity. However, as AOS was continuously added, it provided more surface charges, enhancing the electrostatic repulsion between emulsion droplets and increasing EAI. Additionally, the ESI increased with the addition of AOS, reaching its maximum at a molar ratio of 1:80. This was because AOS caused ferritin to form a layer of oligosaccharides at its interface, thereby increasing its stability to resist oil droplet aggregation and helping to improve adsorption ability of ferritin at the oil–water interface [43,44]. Therefore, a molar ratio of 1:80 (ferritin/AOS) was used in this study.

The EAI of the emulsion after the reaction of ferritin with Zn^2+^ ions was improved (Figure 3b,c), which was same as in previous studies that found that Zn^2+^ ions could increase the EAI of rice protein [45]. This might be because the increase in electrostatic force after the reaction of ferritin with Zn^2+^ ions led to the extension of ferritin, resulting in the denaturation of ferritin, exposing the hydrophobic groups inside ferritin and increasing its surface activity [46]. The adhesion between the protein and the oil–water interface increased, which promoted dispersion of the oil droplets, thereby enhancing the EAI of the emulsion [25]. After adding AOS, ESI of the emulsion was significantly improved. This was because the anionic oligosaccharides could form a protective layer around droplets through electrostatic repulsion with proteins, providing more negative charge, enhancing the electrostatic repulsion between the droplets, preventing them from coalescing after collision, and improving the stability of the emulsion [12].

### 3.4. Zeta-Potential of Emulsions

**Figure 4:**
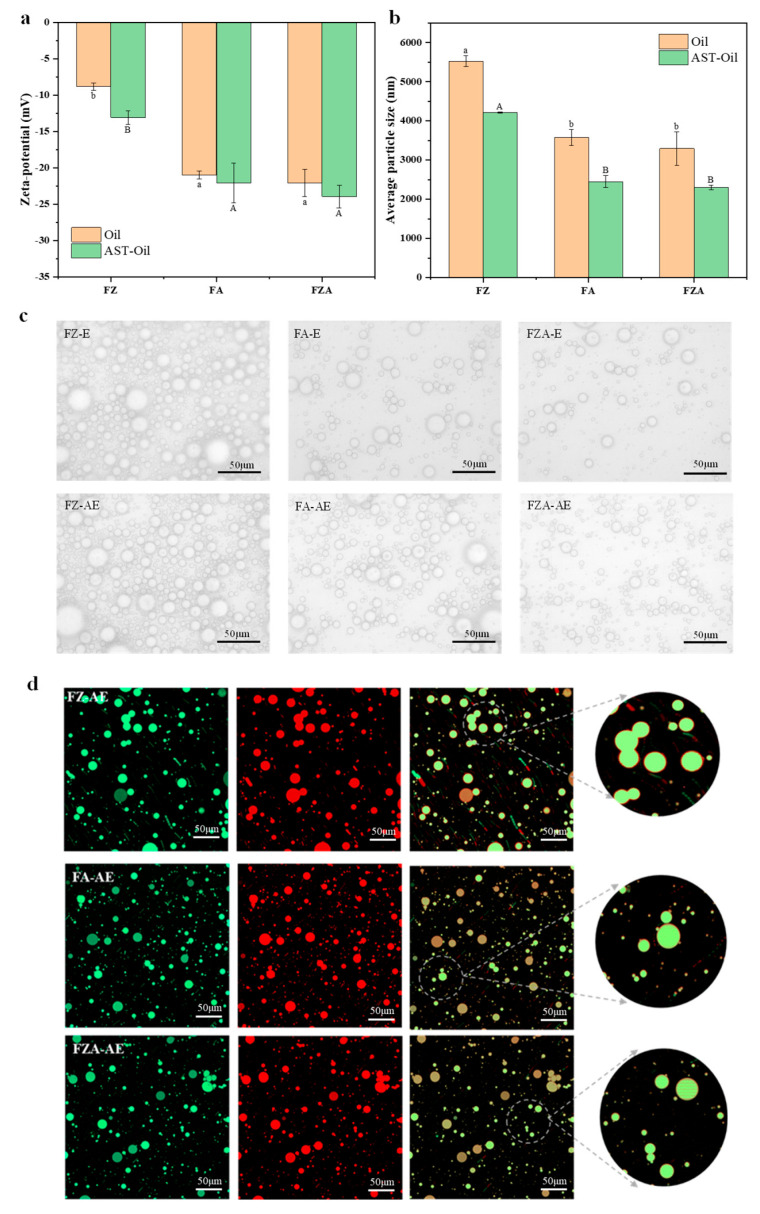
(**a**) Zeta-potential of emulsion stabilized by FZ, FA, and FZA. (**b**) Average particle size of emulsion stabilized by FZ, FA, and FZA. (**c**) Optical micrograph of emulsion stabilized by FZ, FA, and FZA. (**d**) Intelligent high-resolution microscopic image of emulsion where oil droplets were dyed green (**left**) and protein particles were dyed red (**center**) and combined image (**right**). Different letters (a, b/A, B) on the column represent statistically significant (*p* < 0.05).

Zeta-potential measures the charge density on particle surfaces. Typically, emulsions with greater absolute zeta-potentials show enhanced stability [47]. Compared with the FZ-E (−8.816 mV) (Figure 4a), the addition of AOS could reduce zeta-potential of emulsion (−20.967 mV), which might be attributed to AOS possibly increasing the surface charge of oil droplets and providing sufficient electrostatic repulsion for the emulsion. This agreed with previous studies demonstrating that anionic oligosaccharides could enhance emulsion stability, with electrostatic repulsion and steric hindrance being main reasons [12]. When AST was added, the absolute value of the zeta-potential of all emulsions increased. This was because AST altered the composition and charge distribution of the oil–water interface, making the interfacial layer around the oil droplets thicker. This change enhanced the interactions between droplets and consequently increased the absolute value of the emulsion’s zeta-potential.

### 3.5. Morphological Characteristics and Particle Size of Emulsion

In general, larger droplets will make it easier for the emulsion to stratify and thus affect stability and further application of emulsion [48]. The particle size of FA-E and FZA-E (3537 nm, 3297 nm) was smaller than that of FZ-E (5526 nm) (Figure 4b), indicating that AOS made a prominent contribution to improving the stability of ferritin emulsions, which was due to AOS being able to bind to ferritin, promoting formation of a dense interface layer, thereby preventing the interaction and aggregation of droplets [12,19]. When AST was added, the average particle size of droplets was smaller, which might be related to the fact that AST increased viscosity of oil phase and formed a denser network structure of emulsion. In addition, the PDIs of FZ-E, FA-E, and FZA-E were 0.44, 0.31, and 0.29 respectively (Table S1). This indicated that the addition of AOS could improve uniformity of droplet size distribution. When AST was added, the lowest PDI (0.22) observed in FZA-AE suggested that FZA-AE had the most uniform droplet size distribution. The optical microscope results were consistent with the particle size (Figure 4c). The particle sizes of FZ-E and FZ-AE were large, the distribution of droplets was uneven, and there was a droplet aggregation phenomenon. With the addition of AST, the emulsion formed smaller and more homogeneous droplets, the same as the particle size results.

**Figure 5:**
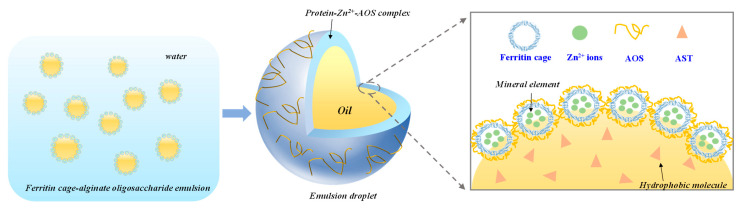
Proposed formation mechanism of emulsions stabilized by ferritin–zinc ion–AOS complex.

To further observe the distribution of oil and protein in emulsion, a high-resolution microscopic fast imaging system was applied to analyze the samples. The oil and protein were dyed green and red, respectively. As shown in Figure 4d, the sample could form spherical droplets with regular shape and uniform distribution. After staining, spherical oil droplets exhibited green fluorescence, while a surrounding red fluorescence indicated protein coverage. This distribution confirmed that FZA adsorbed at the oil–water interface, forming a continuous protein layer that stabilizes emulsion (Figure 5), which is a typical structure of Pickering emulsion [28,49].

### 3.6. Shear Rheological Properties of Emulsion

#### 3.6.1. Frequency Sweep

**Figure 6:**
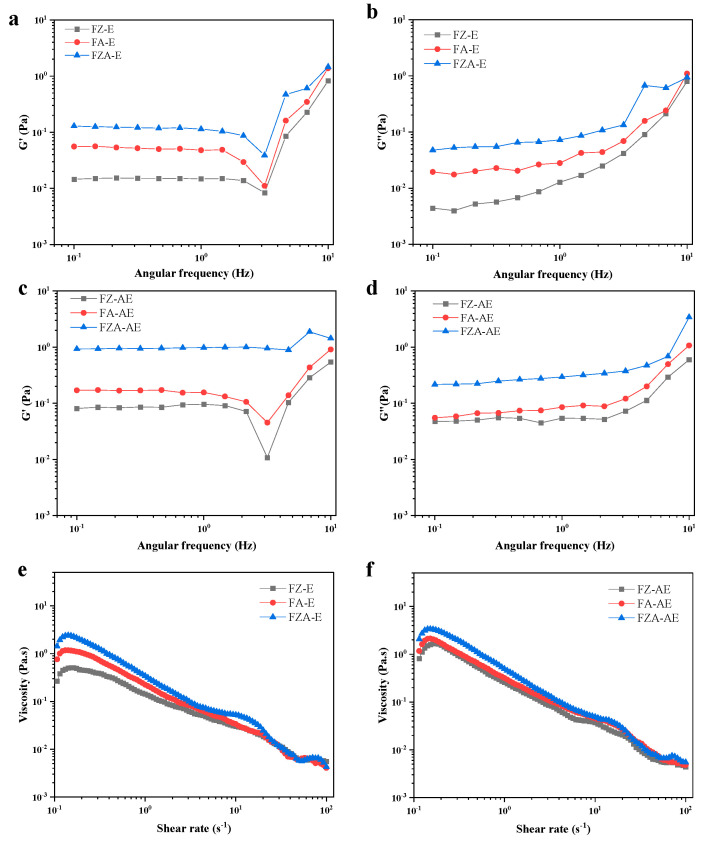
(**a**,**b**) Function of the viscoelastic modulus (G′ and G″) as a function of the frequency (0.1% strain) of emulsion without AST. (**c**,**d**) Function of the viscoelastic modulus (G′ and G″) as a function of the frequency (0.1% strain) of emulsion with AST. (**e**) Apparent viscosity as a function of the shear rate for emulsions without AST and (**f**) emulsions with AST.

Rheology is essential in reflecting properties of emulsions and evaluating their mechanical stability (Figure 6a–d). The G′ (storage modulus) and G″ (loss modulus) of FZA-E was significantly higher than those stabilized by FZ and FA. This indicated that gel strength increased, and possibility of deformation decreased under external force. The formation of this denser network structure was likely attributed to non-covalent interactions, including electrostatic forces and hydrogen bonding, among AOS, Zn^2+^ ions, and ferritin [50,51]. With the addition of AST, the G′ and G″ of emulsion were increased, indicating that addition of AST promoted the formation of a more stable network structure of emulsion [52]. In addition, the G′ was always higher than G″, and with increasing frequency, both G′ and G″ increased, implying the formation of an elastic gel structure [53]. Similar results were observed when ferritin–chitooligosaccharide complexes were used as emulsion stabilizers [19].

#### 3.6.2. Shear Sweep

The apparent viscosity of all emulsions decreased with increasing shear rate, a typical shear-thinning (pseudoplastic) behavior (Figure 6e,f). This phenomenon was attributed to the disruption of droplet flocculation, breakdown of internal structures, and gradual weakening of the gel network at higher shear rates [54,55]. Notably, FZA-stabilized emulsions exhibited higher apparent viscosity than those stabilized by FZ or FA, and the incorporation of AST further increased viscosity.

When fitting the curve using the Ostwald–de Waele model, the same result was obtained (Table 2). If flow behavior index (n) is less than 1, it indicates that sample exhibits shear-dilution behavior. The consistency index (K) reflects emulsion viscosity, with larger K values indicating greater viscosity [19]. In the low-shear-frequency region, the apparent viscosity of FZA-E was higher than FZ-E and FA-E, which was same as the dynamic rheological results. Table 2 shows that the K value of FZA-stabilized emulsion was higher than FZ- and FA-stabilized emulsions, indicating that the addition of Zn^2+^ ions and AOS increased viscosity of emulsion. This was because the electrostatic forces between Zn^2+^ ions, AOS, and ferritin could effectively promote formation of continuous phase [56,57]. When the oil phase contained AST, the K value increased, and the apparent viscosity increased. This might be because AST increased interaction between droplets, which made the emulsion structure more compact, improved deformation resistance of emulsion, and made it more stable. In summary, FZA-AE had the best viscosity, modulus of elasticity and stability against delamination.

### 3.7. Storage Stability of Emulsion

**Figure 7:**
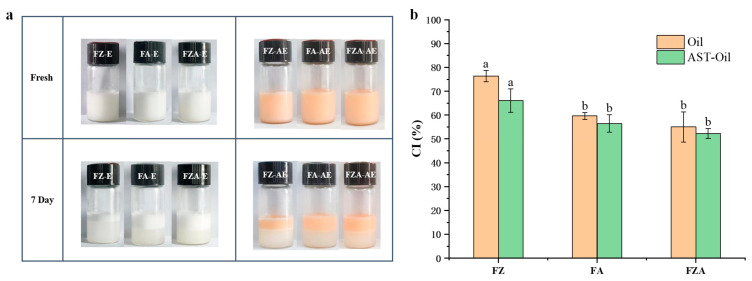
(**a**) The effect of different emulsifiers on the appearance of emulsion. (**b**) CI (%) of different particle-stabilized emulsions after 7 days of storage. Different letters (a, b) on the column represent statistically significant (*p* < 0.05).

Figure 7 shows the visual effects of emulsion stabilized with different emulsifiers, and the storage stabilities of the emulsion were assessed by cream index (CI) [58]. Figure 7a shows the state of the emulsion after just homogenization and after 7 days of storage at 4 °C, respectively. There was no obvious stratification in the freshly prepared emulsion. When AST was added, the color of the sample changed, and no oil phase precipitation was observed, indicating that co-encapsulation of Zn^2+^ ions and AST was realized. When stored for 7 days, the emulsion gradually divided into a serum layer (bottom) and a cream layer (top) [59]. After AOS was added, the CI of the emulsion was reduced, indicating that AOS improved stability of emulsion. The lowest CI and best stability of FZA-AE were attributed to the denser network formed by AOS and Zn^2+^ through non-covalent/electrostatic interactions with ferritin [19].

### 3.8. Encapsulation Efficiency and Stability of AST in Emulsion

#### 3.8.1. Encapsulation Efficiency and Storage Stability of AST

**Figure 8:**
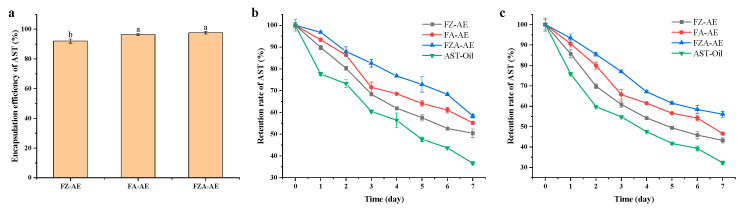
(**a**) Encapsulation efficiency of AST; (**b**) retention rate of AST at 4 °C in darkness and (**c**) at room temperature in darkness. Different letters (a, b) on the column represent statistically significant (*p* < 0.05).

As shown in Figure 8a, the encapsulation efficiencies of AST in FZ-AE, FA-AE, and FZA-AE were 92.04%, 96.41%, and 97.65% respectively. FZA-AE exhibited the highest encapsulation efficiency, which was related to its smaller particle size, more compact interface structure, and the interaction between AOS and ferritin [60]. The higher encapsulation efficiency laid the foundation for the improvement of retention rate under subsequent storage, heat, and light conditions. To evaluate stability of AST, the emulsion containing AST was kept away from light at 4 °C and at room temperature, respectively. Compared with AST in oil, emulsion encapsulation improved retention rate of AST (Figure 8b,c). FZA-AE showed the maximum retention rate, probably because Zn^2+^ ions and AOS worked together to stabilize the emulsion, and improvement of stability provided a strong-enough protective barrier for AST. This was consistent with previous reports that emulsion encapsulation could act as a protective barrier against internal bioactive substances and improve its stability [54,61,62].

#### 3.8.2. Thermal Stability and Photostability of AST

**Figure 9:**
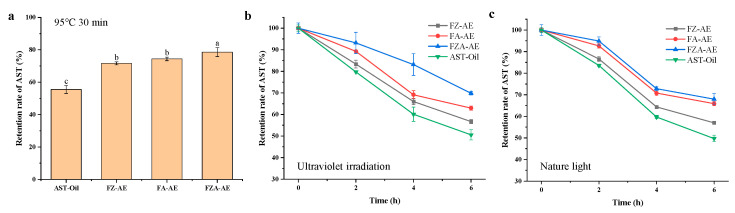
(**a**) The retention rate of AST after heating at 95 °C for 30 min; (**b**) the retention rate of AST under ultraviolet irradiation; (**c**) the retention rate of AST under natural light irradiation. Different letters (a–c) on the column represent statistically significant (*p* < 0.05).

AST is sensitive to oxygen, light and high temperature, so its application is limited [28]. To simulate stability of AST during processing, storage and transportation, the emulsion was protected from high temperature, natural light, and ultraviolet light respectively, and the protection effect of emulsion system on AST was evaluated. As shown in Figure 9a, free AST rapidly decomposed at 95 °C, and the retention rates of AST in emulsion were 71.61% (FZ-AE), 74.38% (FA-AE), and 78.59% (FZA-AE), respectively, which were higher than AST dissolved in oil phase (55.51%). Furthermore, under both ultraviolet and natural light exposure, the emulsion maintained a higher AST retention rate than the oil-dissolved AST. This suggested that AST in the emulsion was well protected under heating and light conditions, thereby significantly improving the storage stability of bioactive compounds [63]. Among them, the retention rate of AST in FZA-AE was the highest, indicating that the emulsion formed by FZA could protect AST more effectively, which might be related to the high stability and apparent viscosity of the FZA-stable emulsion. Secondly, it might be due to the addition of AOS. Owing to its potent radical-scavenging ability, AOS removed free radicals and minimized their interaction with AST [64,65], thereby retarding AST degradation [61,64].

### 3.9. Release Rate of Zn^2+^ Ions and AST

**Figure 10:**
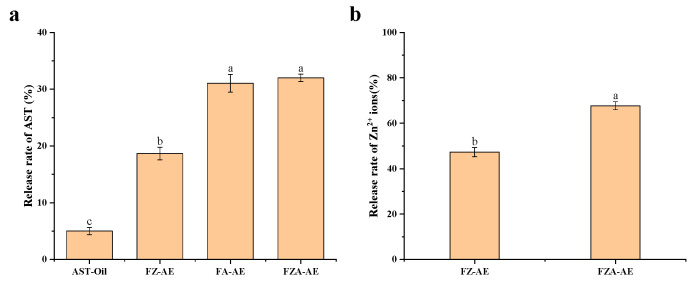
(**a**) Release rate of AST; (**b**) release rate of Zn^2+^ ions. Different letters (a–c) on the column represent statistically significant (*p* < 0.05).

Release rates of FZ-AE, FA-AE, and FZA-AE were 18.66%, 31.06%, and 31.98% respectively, which were higher than oil-dissolved AST (5.01%) (Figure 10a). This was because of the formation of the emulsion’s dense network, which limited oil droplet coalescence during digestion. Consequently, digestive enzymes gained greater access to protein-coated droplet surface, enhancing interfacial protein hydrolysis and ultimately leading to droplet breakdown. Therefore, more AST was released, increasing the release rate [66]. The release rate of AST in FA-AE and FZA-AE was higher than that in FZ-AE, which might be due to the higher interface stability conferred by AOS, making the oil droplets less prone to aggregation during digestion, thereby increasing the contact area with digestive enzymes. Similarly, the release rate of Zn^2+^ ions in FZA-AE (67.69%) was significantly higher than that in FZ-AE (47.31%) (Figure 10b), which was attributed to the smaller droplet size and stronger interface protection layer in FZA-AE, facilitating the release of Zn^2+^ ions after the iron protein shell was hydrolyzed by protein enzymes.

### 3.10. Comparison with Previous Work on Similar Ferritin-Stabilized Emulsion System

Although previous studies have reported the use of ferritin to encapsulate various active substances in emulsion systems, most of these studies focused on the encapsulation of a single component or simple hydrophobic active substances [59,67,68], without involving the simultaneous delivery of hydrophobic bioactive substance and mineral element. In this study, mineral element (Zn^2+^ ions) was introduced into the ferritin–oligosaccharide complex, and a ferritin–zinc ion–AOS complex was constructed as an emulsifier to successfully form multilayer emulsions, achieving co-encapsulation of AST (in the oil phase) and Zn^2+^ ions (in the ferritin cavity), and improving the stability and release rate of AST and Zn^2+^ ions. In addition to this co-delivery capability, the presence of Zn^2+^ ions was also found to be able to regulate the interface properties of the emulsion. Specifically, Zn^2+^ ions enhanced the electrostatic, hydrogen bond, and hydrophobic interactions between ferritin and AOS, thereby forming a denser interface layer with a higher viscoelastic modulus and smaller droplet size. Therefore, the main contribution of this study lies in the establishment of a ferritin-based emulsion platform for the co-delivery of hydrophobic bioactive substance and mineral element. The regulatory role of Zn^2+^ ions provided another mechanistic insight, which might offer a reference for enhancing the design of mineral-containing emulsion systems.

## 4. Conclusions

The unique cage-like structure of ferritin allows ferritin-based emulsions to simultaneously encapsulate and deliver diverse bioactive ingredients. In this study, Zn^2+^ ions were encapsulated into the ferritin, and FZA was constructed as an emulsifier to successfully form multilayer emulsions, achieving co-encapsulation of AST (in the oil phase) and Zn^2+^ ions (in the ferritin cavity). Zn^2+^ ions acted as a bridge to significantly enhance the multi-force interactions (electrostatic, hydrogen bonding, and hydrophobic) between ferritin and AOS, leading to a denser interfacial network. This resulted in superior emulsifying stability, protection against environmental stresses, and enhanced stability and in vitro release rate of Zn^2+^ ions and AST. This study demonstrated simultaneous delivery of both mineral ions and hydrophobic bioactive ingredients, underscoring the promise of ferritin-stabilized emulsions as dual-compartment carriers and providing a foundation for further research on future exploration of ferritin-based emulsions in multi-component bioactive delivery.

## Figures and Tables

**Table 1:** The relative interaction force strength (%) of FZ, FA, and FZA.

Interaction Forces	FZ	FA	FZA
Electrostatic interaction	10.95 ± 0.59 ^b^	6.73 ± 1.06 ^c^	14.23 ± 1.59 ^a^
Hydrogen bonding	7.19 ± 1.18 ^b^	9.92 ± 1.62 ^b^	13.54 ± 2.08 ^a^
Hydrophobic interaction	1.71 ± 0.59 ^c^	8.15 ± 0.61 ^b^	15.62 ± 2.08 ^a^

Different letters (a–c) on the column represent statistically significant (*p* < 0.05).

**Table 2:** Flow consistency (K) and flow index (n) of the emulsions.

Samples	Flow Consistency (K)	Flow Index (n)
FZ-E	0.1496	0.2590
FA-E	0.2300	0.1253
FZA-E	0.3558	0.0499
FZ-AE	0.2512	0.0784
FA-AE	0.3144	0.0764
FZA-AE	0.4549	0.0669

## Data Availability

The original contributions presented in this study are included in the article/Supplementary Materials . Further inquiries can be directed to the corresponding authors.

## Supplementary Materials

The following supporting information can be downloaded at https://www.mdpi.com/article/10.3390/foods15101666/s1. Table S1: The PDI of the emulsions.
