# Supplementary material for: Ferritin Cage–Alginate Oligosaccharide-Stabilized Emulsion for Co-Carriage and Protection of Zinc Ion and Hydrophobic Molecule
Source: Foods. 2026 May 11;15(10):1666. doi: 10.3390/foods15101666 (PMC13205369; doi:10.3390/foods15101666)
Supplement: Supplementary file 1 [file foods-15-01666-s001.zip › foods-4219421-supplementary.pdf]

Table S1. The PDI of the emulsions.

Samples	PDI
FZ-E	0.47 ± 0.02^{a}
FA-E	0.31 ± 0.03^{c}
FZA-E	0.29 ± 0.02^{cd}
FZ-AE	0.35 ± 0.02^{b}
FA-AE	0.27 ± 0.02^{d}
FZA-AE	0.22 ± 0.01^{e}

Different letters (a–e) on the column represent statistically significant ($p < 0.05$).
